# Mechanisms, therapeutic uses, and developmental perspectives of redox-active thiomolybdates

**DOI:** 10.1016/j.redox.2025.103846

**Published:** 2025-08-27

**Authors:** Yihan Wu, Khalid S. Alotaibi, Kevin Yu, Tom Durham, Felipe Dal-Pizzol, Mervyn Singer, Alex Dyson

**Affiliations:** a Institute of Pharmaceutical Science, King's College London, London, UK; b Bloomsbury Institute of Intensive Care Medicine, Division of Medicine, University College London, London, UK; c King Faisal Specialist Hospital and Research Centre, Riyadh, Saudi Arabia; d NIHR Southampton Clinical Research Facility and NIHR Southampton Biomedical Research Centre, University Hospital Southampton NHS Foundation Trust, UK; e Laboratory of Experimental Pathophysiology, University of Southern Santa Catarina, Criciúma, Brazil; f Centre for Pharmaceutical Medicine Research, King's College London, London, UK

**Keywords:** Hydrogen sulfide, Tetrathiomolybdate, Mitochondria, Cancer, Copper chelator, Reperfusion injury, Wilson's disease

## Abstract

Redox-active, copper-chelating thiomolybdates are a family of metal-based therapeutics used to treat copper toxicity in animals and Wilson's disease in humans, and studied in other indications including cancer, inflammatory and fibrotic conditions. Thiomolybdates act through multiple mechanisms including copper chelation, redox regulation (e.g., superoxide dismutase inhibition), and modulation of inflammation. We and others have also identified thiomolybdates as slow-release sulfide donors that inhibit mitochondrial respiration, limit mitochondrial reactive oxygen species (ROS) production, augment antioxidant reserve capacity, and confer organ- and whole-body protection in non-clinical models of reperfusion injury. Here we review the rich history of the thiomolybdate drug class, focusing on their activity across multiple pathologies, utility in non-clinical and clinical settings, accepted and proposed mechanisms of action, developmental perspectives, and limitations. Context-specific use of thiomolybdates support their development as either first-in-class or next generation therapeutics across several disease areas. Dosing and route of administration differentiate the utility of thiomolybdates as either copper chelators (oral administration over several weeks) or sulfide donors (acute intravenous use). Further work is however required to understand the impact of both opposing and additive mechanisms of action. Examples include reduction of ROS generation versus superoxide dismutase inhibition in oxidative pathologies, and the opposing angiogenic effects of copper chelation and sulfide bioavailability in the tumor microenvironment.

## Graphical abstract

**Figure:**
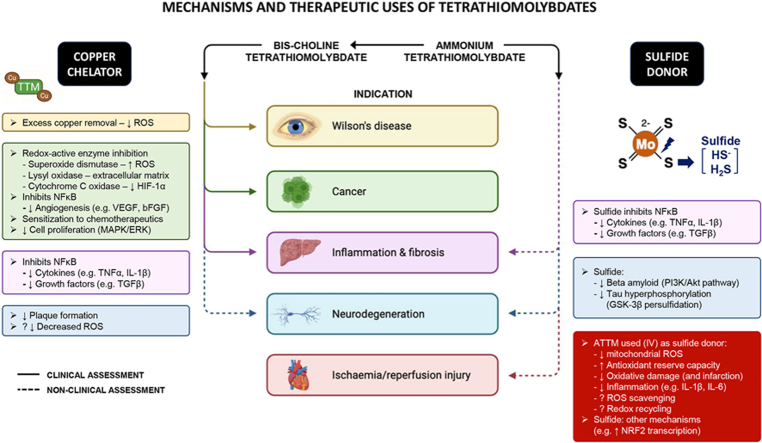

## Abbreviations

Alb: Albumin
AMPK: AMP-activated protein kinase
Atox1: Antioxidant-1 chaperone
ATP7A: Copper-transporting ATPase-1
ATP7B: Copper-transporting ATPase-2
ATTM: Ammonium tetrathiomolybdate
ATTT: Ammonium tetrathiotungstate
AUC: Area under curve
BCTTM: Bis-choline tetrathiomolybdate
bFGF: Basic fibroblast growth factor
BRAF: Proto-oncogene B-Raf/v-Raf murine sarcoma viral oncogene homolog B
CcOx: Cytochrome *c* oxidase
CCS: Copper chaperone protein
C_MAX_: Maximum plasma concentration
COX17: Cyclooxygenase 17
Cp: Ceruloplasmin
CTR1: Copper transporter 1
ECM: Extracellular matrix
EPC: Endothelial progenitor cell
ERK: Extracellular signal-regulated kinase
FDA: Food and Drug Administration
GPx: Glutathione peroxidase
GSH: Glutathione (reduced form)
GSSG: Glutathione (oxidized form)
GSSH: Glutathione persulfide
H_2_O_2_: Hydrogen peroxide
H_2_S: Hydrogen sulfide gas
H_2_S_2_: Hydrogen persulfide
H_2_Se: Hydrogen selenide gas
HIF-1α: Hypoxia-inducible factor-1α
HS^−^: Hydrosulfide anion
HSe^−^: Hydroselenide anion
IL-1β: Interleukin-1β
IL-2: Interleukin-2
IL-6: Interleukin-6
IRI: Ischemia/reperfusion injury
LEC: Long-Evans (rat) with a cinnamon-like coat
LOX: Lysyl oxidase
ΔΨm: Mitochondrial membrane potential
mTOR: Mammalian target-of-rapamycin
MAPK: Mitogen-activated protein kinase
MeCN: Acetonitrile
MEK1: Mitogen-activated protein kinase kinase-1
Na_2_S: Sodium sulfide
NADP: Nicotinamide adenine dinucleotide phosphate (oxidized form)
NADPH: Nicotinamide adenine dinucleotide phosphate (reduced form)
NF-κB: Nuclear factor-κB
NRF2: Nuclear factor erythroid 2-related factor 2
O_2_^•^: Superoxide
•OH: Hydroxyl radical
PI3K/Akt: Phosphatidylinositol 3-kinase/protein kinase B
PK/PD: Pharmacokinetic/pharmacodynamic
RAS: Rat sarcoma
ROS: Reactive oxygen species
SCLY: Selenocysteine gamma-lyase
SOD1: Cu/Zn superoxide dismutase
TNF-α: Tumor necrosis factor-α
TGFβ: Transforming growth factor-β
TTM: Tetrathiomolybdate
VEGF: Vascular endothelial growth factor
Vfb: Vemurafenib

## 1 Introduction

The pursuit of metal-containing medicines (metallotherapeutics) has continued since the discovery of pioneering therapies for syphilis such as mercury and arsenic [1]. Others followed, including iron, zinc, silver-based antimicrobial compounds, the antidiarrheal agent, bismuth subsalicylate, and platinum-based anti-cancer agents such as cisplatin [[2], [3], [4], [5]]. However, the number of licensed compounds remains relatively limited compared to the myriad small molecular entities, biologics and other advanced therapies now being approved [6].

Rationale-based metallotherapeutic drug design requires a detailed understanding of both the therapeutic target and the mechanism(s) of action of the putative agent. Broadly, these mechanisms include (i) covalent binding to biomolecules, (ii) enzyme inhibition or catalytic enhancement, (iii) participation in redox reactions and (iv) ligand release from metal-containing prodrugs, reviewed extensively elsewhere [7]. Metals are used therapeutically in a variety of advanced formulations (e.g., gold nanoparticles), and frequently utilized for non-pharmacological purposes (e.g., as radiographic contrast agents). In this review we focus on the thiomolybdate drug class, describing its unique and complex history, molecular mode(s) of action, novel mechanisms, limitations, and developmental perspectives.

## 2 Tetrathiomolybdate: synthesis and nomenclature

**Fig. 1:**
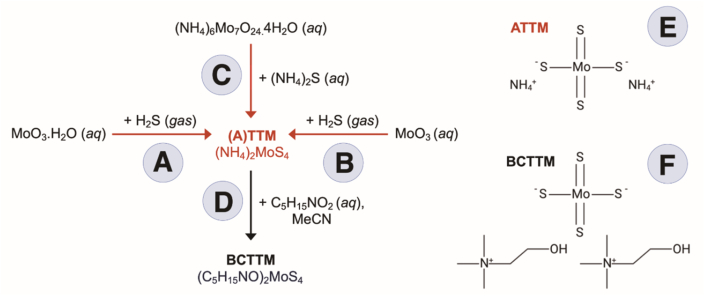
**Synthesis and structure of thiomolybdates.** A and B show synthesis of tetrathiomolybdate (TTM) using oxomolybdate solutions and hydrogen sulfide gas (H_2_S). C shows the synthetic route of ammonium tetrathiomolybdate (ATTM) using solid state precursors in aqueous solutions. D illustrates cation exchange using excess aqueous choline hydroxide to generate BCTTM. The structures of the two major salts of TTM (ammonium TTM; ATTM, and bis-choline TTM; BCTTM) are shown in E and F, respectively. MeCN, acetonitrile.

The noted Swedish chemist, Jans Jacob Berzelius, documented the first synthesis of thiomolybdates in his seminal work *‘On the sulfur salts’* [8]. He created 120 arsenic and molybdenum sulfur compounds, including the first tetrathiomolybdate (TTM; MoS_4_^2−^). The synthetic route involved bubbling aqueous oxomolybdate solutions (Fig. 1A and B) with hydrogen sulfide gas (H_2_S). The reaction was performed at elevated pressures and within closed systems to prevent leakage of H_2_S, a respiratory toxicant in excess. The resulting compounds, of which Berzelius generated numerous salt forms, consisted of a molybdenum core coordinated to four covalently bound sulfur atoms. This work was followed up in 1884 with generation of thiomolybdates in both amorphous and crystalline forms using more contemporary approaches with solid state precursors [9]. Here, aqueous ammonium sulfide and ammonium heptamolybdate tetrahydrate were combined to generate the ammonium salt of TTM ([NH4]_2_MoS_4_^2−^; herein ATTM, Fig. 1C), that reduced the potential for H_2_S toxicity.

This century saw a new generation of thiomolybdates; principally, a second generation bis-choline TTM salt [10,11]. This salt, identified chronologically as decuprate, ATN-224, WTX-101 and, most recently, ALXN-1840, is referred to herein as bis-choline TTM (BCTTM). While BCTTM exhibits a similar pharmacological profile to its parent ATTM salt, the organic cation contributes greater stability [11], likely attributed to the presence of a robust hydrogen bond network. ATTM is used as a precursor for the synthesis of BCTTM, with cation exchange enabled by reaction of one equivalent of ATTM with an excess of aqueous choline hydroxide solution (Fig. 1D) [12]. Herein, thiomolybdates are referred to collectively as TTM unless a specific salt form is specified. Further information on ATTM chemistry has been reviewed in detail elsewhere [13].

**Fig. 2:**
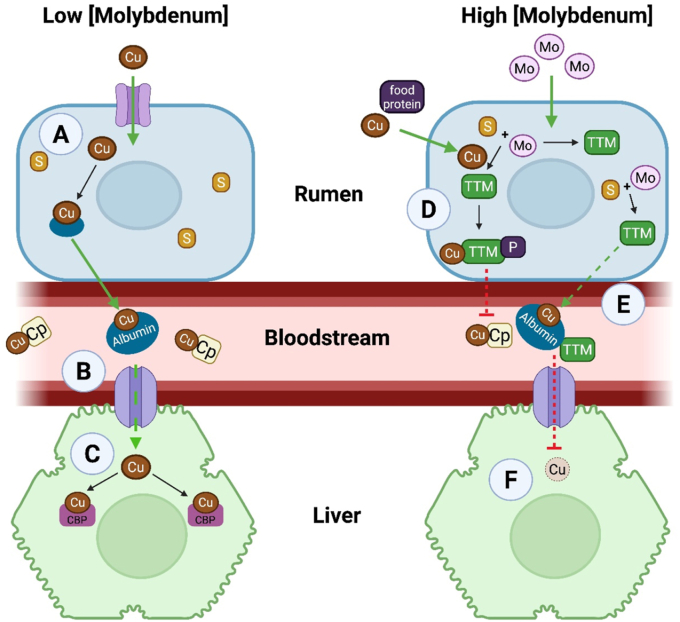
**The endogenous thiomolybdate hypothesis.** In healthy conditions, dietary copper (Cu) is absorbed by ruminal enterocytes (A) and binds to copper carrying proteins in blood (B). Protein-bound copper is delivered to liver hepatocytes (C) which binds to copper binding proteins (CBP) and is directed towards ceruloplasmin synthesis and copper storage. If ruminal molybdenum concentrations are increased, it reacts with sulfur to form endogenous thiomolybdates (D); copper is subsequently sequestered in a copper-tetrathiomolybdate-protein complex. Thiomolybdates also reach the systemic circulation and chelate plasma copper (E), which decreases copper levels in hepatocytes (F). Alb, albumin; Cp, ceruloplasmin; Cu, copper; Mo, molybdenum; P, protein; S, sulfur; TTM, tetrathiomolybdate.

Thiomolybdates can be produced endogenously. Initial observations came from cattle grazing on ‘teart’ pastures in England's West Country in the early 20th century, where molybdenum-rich soil caused ‘scouring’ in cattle – severe diarrhoea and reduced milk yield – that was reversible with copper sulfate [14,15]. Similar symptoms appeared in ewes fed molybdate- and sulfate-rich diets [[16], [17], [18]]. These animals showed significantly reduced liver copper levels [16,19,20]. In healthy conditions with normal molybdenum levels, copper is absorbed in the rumen and binds to plasma proteins such as ceruloplasmin (40–70 %), albumin (10–15 %), and transcuprein/macroglobulin (10–15 %) (Fig. 2A and B). It is then transported mainly to the liver for storage and ceruloplasmin synthesis [21] (Fig. 2C).

The endogenous thiomolybdate hypothesis suggests excess ruminal molybdenum reacts with sulfide to form thiomolybdates (Fig. 2D). These bind copper and proteins, forming ‘tripartite’ Cu-thiomolybdate-protein complexes [22]. These complexes limit dietary copper absorption and sequester plasma copper, reducing hepatic uptake and systemic levels [[22], [23], [24], [25]] (Fig. 2E and F). This copper-chelating property of thiomolybdates was used to treat copper toxicity in sheep [26] and, subsequently, Wilson's disease in humans [27].

## 3 Pharmacokinetics

To our knowledge, information on the pharmacokinetics of ATTM [28] (DrugBank) in humans is unavailable in the public domain. In a canine study, ATTM exhibited low and highly variable oral bioavailability (21 %) [29]. This was primarily due to poor gastrointestinal absorption and, in some cases, emesis. The terminal half-life of ATTM by either oral or intravenous administration was similar (∼27 h) in this species. A clinical study with BCTTM reported a half-life of 34 h, considered supportive of once-daily dosing [11]. The comparative molecular stability of BCTTM also causes fewer gastrointestinal side effects, such as sulfur eructation. Co-administration with proton pump inhibitors increases the C_MAX_ and AUC of BCTTM [10,11] and provides a further means to prevent acid-hydrolysis of the MoS_4_ complex. Taken together, these advantages make BCTTM a more promising candidate than ATTM for depletion of copper.

## 4 Potential therapeutic indications and underlying mechanisms

**Fig. 3:**
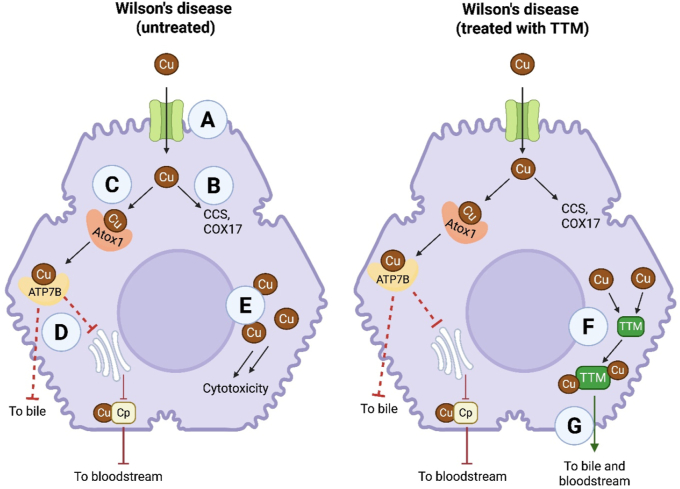
**Disrupted copper homeostasis in Wilson's disease and treatment with tetrathiomolybdate.** Following cellular uptake by copper transporter 1 (CTR1) (A), free copper (Cu) binds rapidly to chaperones e.g., copper chaperone protein (CCS) and cyclooxygenase 17 (COX17) (B). Copper also binds to the antioxidant-1 chaperone (Atox1) (C), which delivers copper to the ATP7B (and ATP7A) transporter proteins. ATP7B, responsible for biliary excretion of copper and its incorporation into ceruloplasmin (Cp), is disrupted by mutations to the ATPase (D), resulting in accumulation of intracellular copper (E) and cytotoxicity. Tetrathiomolybdate sequesters free (and metallothionein-bound) copper (F), with the resultant copper-TTM complex being excreted (G). ATP7B, copper-transporting ATPase 2; Cp, ceruloplasmin; Cu, copper; TTM, tetrathiomolybdate.
  Reaction of TTM with Cu-Atox1 forms a stable complex that can be isolated via size-exclusion chromatography [37]. X-ray crystallography revealed twelve Cu-Atox1 molecules organized into four trimers, each forming a complex with one TTM molecule and four copper ions. The resulting structure has the stoichiometry [TTM][(Cu)(Cu-Atx1)]_3_ and forms an S_6_Cu_4_MoS_4_ cluster composed of four copper ions, one [MoS_4_]^2-^ unit, and three pairs of Atox1 cysteine residues. Mechanistic studies indicate that TTM suppresses copper trafficking by targeting domains involved in the activation and secretion of copper-dependent enzymes [37].
  Non-clinical assessment of TTM for treating copper toxicity has primarily been performed in LEC rats (Long-Evans rats with a cinnamon-like coat) which exhibit a rodent form of Wilson's disease [[38], [39], [40], [41], [42]]. This strain possesses a genetic defect, homologous to the human ATP7B mutation, with hepatic copper accumulation ultimately leading to hepatitis and liver failure [43]. This model has consistently demonstrated the ability of ATTM or BCTTM to deplete excess hepatic copper [38,42,44].
  ATTM has been trialed clinically as a treatment for Wilson's disease through a series of studies, initially conducted by Brewer and colleagues [27,[45], [46], [47], [48]] (Supplementary Table 1). These studies demonstrate the ability of ATTM to treat the condition and highlight advantages over traditional treatments such as penicillamine and trientine [[47], [48], [49]]. ATTM prevented neurological deterioration, which can often be exacerbated by these other treatments, resulting in irreversible damage due to mobilization of liver copper stores that increase free copper in the brain [[47], [48], [49]]. ATTM was generally well-tolerated across these trials, with the most notable side effects being mild anemia/leukopenia, and elevated aminotransferases; these were both exposure-related and reversible [[45], [46], [47]]. More recent clinical trials have been performed using the more stable BCTTM. Positive results were reported in a Phase III trial [50], although a more recent study investigating the effects of BCTTM in healthy volunteers and Wilson's disease patients found that BCTTM could reduce intestinal copper uptake by a clinically significant degree, although the drug did not facilitate biliary copper excretion [51]. Further studies are warranted to ascertain the therapeutic utility of TTM for Wilson's disease.
(ii) Cancer
  The dependence on copper by cancer cells is well-established, with elevated copper levels evident in various experimental cancer pathologies [[52], [53], [54], [55]] and in patients [[56], [57], [58]]. Following tumor reduction or removal, circulating copper levels return to normal [59]. This has led to the exploration of copper chelators as potential anti-cancer agents [60]. However, excess copper can have opposing effects [61]. While copper augments angiogenesis and cell proliferation, it is also associated with autophagy, mitophagy, apoptosis and ROS-induced toxicity [55,59,62,63]. In non-clinical studies, both ATTM and BCTTM, deployed either as an adjunct or monotherapy, showed utility against various tumor subtypes, including breast cancer, ovarian cancer, head and neck squamous cell carcinoma, non-small cell lung cancer and liver cancer [53,[64], [65], [66], [67], [68], [69], [70], [71], [72], [73], [74], [75]] Multiple molecular modes of action have been postulated (Fig. 4, Fig. 5).
  • *Effect of TTM on angiogenesis*
    The association between copper and angiogenesis was first reported in 1980 through demonstration of augmented bovine endothelial cell migration induced by copper salts [76]. Restriction of nutrient supply through inhibition of angiogenesis was initially considered the primary means by which copper depletion impedes cancer development [65,71,77]. Copper promotes angiogenesis through modulation of proangiogenic growth factors including vascular endothelial growth factor (VEGF), basic fibroblast growth factor (bFGF) and angiogenin [78]. Both VEGF and bFGF are required for proliferation and migration of endothelial cells, a process stimulated by copper in human cell cultures [79]. These growth factors are usually over-expressed in cancer [80]. Depletion of copper with TTM inhibits VEGF and bFGF production, primarily through nuclear factor-κB (NF-κB) regulation (Fig. 4A and B), resulting in reduced angiogenesis and tumor growth [70,71].
  • *Redox-active enzyme inhibition*
    TTM can modulate the activity of copper-containing enzymes that include ceruloplasmin, ascorbate oxidase, lysyl oxidases (LOX), SOD1, and CcOx [37,81]. The function of some of these enzymes are dysregulated in multiple cancer types [61,82].
    LOX secreted by cells operates extracellularly to remodel the extracellular matrix (ECM) by catalyzing the crosslinking of collagen and elastin [64]. LOX requires copper for the formation of quinone cofactors, and for facilitating the oxidative deamination reactions that these enzymes catalyze [83]. Since the ECM is a crucial component for the development of vascular networks and the premetastatic niche, inhibiting LOX activity is an additional mechanism by which TTM can attenuate angiogenesis and cancer metastasis [64] (Fig. 4C).
    TTM downregulates the activity of SOD1 [35,84], a cytosolic enzyme that detoxifies superoxide (O_2_^•-^) to hydrogen peroxide (H_2_O_2_) and molecular oxygen. The copper-zinc metal center of SOD1 is critical for the two-step dismutation reaction to occur, as the redox activity of copper is required for the alternate reduction and oxidation of the active-site copper [85]. In cancer cells, inhibition of SOD1 (Fig. 4D) leads to elevated superoxide levels, exacerbating oxidative damage and promoting apoptosis [86]. TTM has been proposed to inhibit SOD1 by removing copper from the enzyme [37] although the reaction chemistry has yet to be fully resolved.
    CcOx, the terminal oxidase of the mitochondrial electron transport chain, utilizes three copper ions arranged into two redox centers (CuA and CuB) [87]. CcOx catalyzes transfer of electrons from reduced cytochrome *c* to molecular oxygen. This contributes to the proton gradient that drives ATP synthesis and is therefore crucial for oxidative phosphorylation and cellular metabolism [88]. In the hypoxic tumor microenvironment, there is an increased metabolic reliance on glycolysis rather than oxidative phosphorylation that persists even when oxygen is available (the Warburg effect) [89]. Notwithstanding glycolytic ATP production being less efficient than oxidative phosphorylation, aerobic glycolysis is considered advantageous to cancer cells due to: 1) removal of allosteric inhibition of phosphofructokinase by ATP, allowing uncontrolled glycolysis to facilitate cancer cell growth, 2) provision of maintained metabolism despite variations in oxygen availability in hypoxic/anoxic microenvironments, 3) promotion of an acidic environment that stimulates cancer growth and metastasis, and 4) an increase in reducing equivalents and antioxidant reserve capacity; reviewed in Ref. [90]. While the Warburg effect may be beneficial in cancer cells with an intact respiratory chain, its disruption through CcOx inhibition appears detrimental. Experimental studies showed that TTM-induced CcOx inhibition could decrease mitochondrial membrane potential (ΔΨm), ATP levels and cell proliferation [67,91] (Fig. 4E). Interestingly, the metabolic phenotype (and flexibility) of tumors may be modulated (at least in part) by copper bioavailability in the tumor microenvironment, thus providing a direct mechanistic target that is TTM-sensitive [91]. CcOx inhibition further impacts (potentially through elevated oxygen tensions) upon cancer development through stabilization of hypoxia-inducible factor-1α (HIF-1α) [67], a prominent pro-angiogenic transcription factor commonly upregulated in the hypoxic tumor microenvironment [92]. HIF-1α can also be stimulated by exposure to copper, with reduced copper availability providing a further means of inhibiting its transcription [93].
  • *Other mechanisms of TTM activity*
    Experimentally, TTM is an effective adjuvant alongside traditional anticancer agents including doxorubicin [66] and cisplatin [94]. While the potentiation of doxorubicin efficacy has been attributed to an increase in ROS generation and cancer cell apoptosis [66], the synergy between TTM and cisplatin has uncovered a novel mechanism (Fig. 5). As noted above, TTM forms complexes with various proteins involved in intracellular copper transport, such as Atox1 and the ATP7A/B efflux transporters [37,[94], [95], [96]]. Formation of these complexes prevents binding of cisplatin to these proteins (Fig. 5A), thus reducing its cellular efflux, a prominent resistance mechanism in cancer cells [94] and augmenting its pharmacological activity (Fig. 5B). In further support of this mechanism, inhibition of copper-transporting chaperones (e.g., Atox1) perturbs growth and proliferation of cancer cells, with minimal impact on healthy cells [66,97,98].
    The mitogen-activated protein kinase/extracellular signal-regulated kinase (MAPK/ERK) signaling pathway occurs through high-affinity binding interactions between copper and MAP kinase kinase-1 (MEK-1) [99,100]. Copper depletion by TTM, as well as pharmacological interference of Atox1, inhibits phosphorylation by MEK-1 (Fig. 5C), thereby suppressing MAPK/ERK-induced cell proliferation [99] (Fig. 5D). Inhibitory effects on this pathway stall the development of BRAFV600E-driven tumors in animal models, which arise from the oncogenic V600E mutation in BRAF kinase (upstream of MEK-1). Inhibiting the copper-MEK interaction (and the MAPK pathway), appears to be the key driver for efficacy of the combination therapy of TTM and vemurafenib (Vfb), a BRAF-kinase inhibitor (Fig. 5E) [96,100]. This finding also highlights the potential of TTM-inclusive combination therapy to reduce treatment resistance in BRAFV600E-associated cancers that are known to arise from reactivation of the MAPK pathway [99,101,102].
  • *Clinical trials using TTM in cancer*
    The translation of TTM to the clinical setting as an anti-cancer agent has produced mixed results (Supplementary Table 2) [11,[103], [104], [105], [106], [107], [108], [109], [110], [111], [112]]. A notable difference between the Wilson's disease and cancer trials is the duration of treatment being comparatively longer (beyond 2 years) in some cancer patients [111]. Notwithstanding this, administration of either ATTM or BCTTM is still generally well-tolerated. Adverse events reported in cancer trials include both hematological (e.g., leukopenia, neutropenia, anemia) and non-hematological (e.g., sulfur eructation and fatigue) complications (Supplementary Table 2). The hematological effects are comparable to those described in the Wilson's disease studies, and similarly reversible through dose reduction.
    TTM monotherapy was largely ineffective at preventing disease progression in advanced cancer patients [106,107]. This was attributed to the presence of a larger pool of angiogenic factors within advanced human tumors, some of which are unaffected by copper availability [52]. It has been proposed that TTM-induced copper modulation should be maintained for between 60 and 90 days before the anticancer response can be assessed to allow time for the tumor microenvironment to become copper deficient. Therefore, advanced cancer patients may not be suitable for TTM treatment due to an increased risk of disease progression during this induction period [104]. Focus has thus switched to assessing the effects of TTM on early, micro-metastatic cancers, or to its use as a relapse prevention strategy in patients in remission, but with a high risk of recurrence [52,105,108]. Two clinical trials that utilized TTM to circumvent breast cancer relapse in stage II triple-negative breast cancer patients, as well as those in stage III/IV, demonstrated that copper depletion promotes tumor dormancy with the potential to attenuate metastatic development [105,108]. This may occur through inhibition of two key components of the tumor microenvironment, namely development of endothelial progenitor cells (EPCs) and modulation of LOX enzyme activity. A Phase 1b study, followed by a future randomized Phase 2 trial (currently recruiting), will assess whether adding three years of ATTM to the standard six-month regimen of capecitabine and pembrolizumab improves outcomes in high-risk triple-negative breast cancer patients [113].

(i) Wilson's disease
  Wilson's disease is a rare autosomal recessive genetic disorder affecting approximately 1 in 30,000 people, caused by mutation of the *ATP7B* gene [22]. *ATP7B* encodes the transmembrane copper-transporting ATPase 2 (ATP7B) which is responsible for biliary excretion of excess copper from the liver, and incorporation of copper into the ceruloplasmin protein [30]. Both these processes ensure that free copper within the liver (the central organ for copper homeostasis) is kept to a minimum [31,32]. Dysregulated copper accumulation causes redox stress, gene expression modification, protein inhibition, and mitochondrial dysfunction [33]. Clinical manifestations of Wilson's disease include liver dysfunction (e.g., chronic hepatitis and cirrhosis), neurological abnormalities (e.g., dystonia, tremor, gait disturbances), corneal Kayser-Fleischer rings and renal failure [31].
  The cellular basis of Wilson's disease and its treatment with TTM is shown in Fig. 3. Copper is taken up into hepatocytes via copper transporter 1 (CTR1) [34] (Fig. 3A). Once inside the cell, copper is rapidly bound to metallothionein (MT) or to chaperones (Fig. 3B) such as copper chaperone protein (CCS) and cyclooxygenase-17 (COX17). These chaperones coordinate transport of copper to Cu/Zn superoxide dismutase (SOD1) and cytochrome *c* oxidase (CcOx) enzymes, respectively [30]. Copper is also taken up by the antioxidant-1 (Atox1) metallochaperone (Fig. 3C) that delivers copper to the ATP7B and ATP7A transporter proteins. Biliary excretion of copper by ATP7B and its incorporation into ceruloplasmin is disrupted by mutations to the ATP7B protein (Fig. 3D). This is responsible for intracellular copper accumulation within the liver and other organs, most notably the brain [30]. Excess copper causes injury through production of reactive oxygen species (ROS), such as the generation of hydroxyl radicals (^•^OH) from hydrogen peroxide via Fenton-like reactions (Fig. 3E) [35]. TTM circumvents this toxicity by sequestering free (and MT-bound) copper (Fig. 3F), with resulting biliary excretion of the copper-TTM complex (Fig. 3G) [36].

**Fig. 4:**
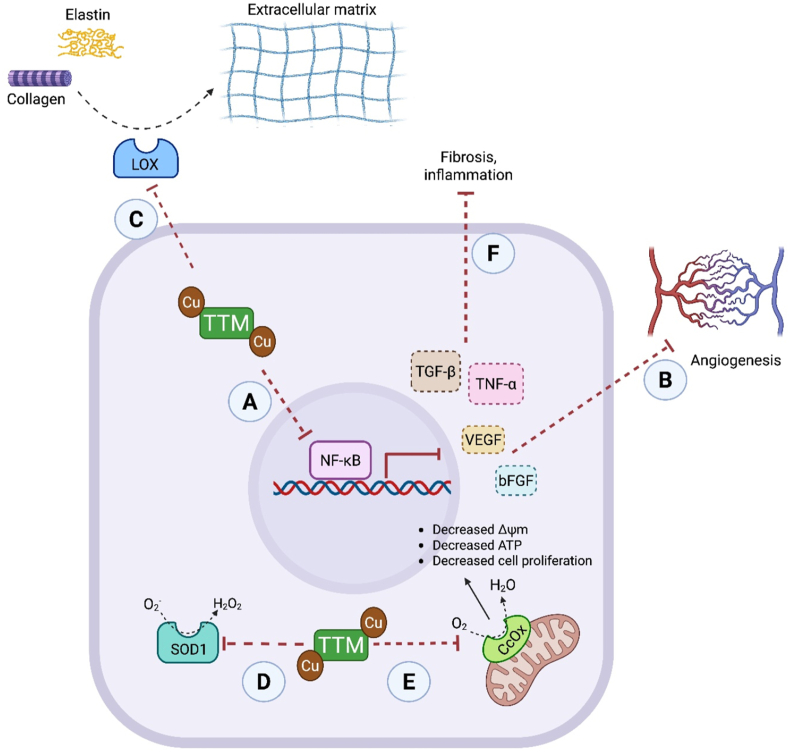
**Mechanisms for disruption of cancer, inflammatory and fibrotic pathologies by TTM-mediated copper depletion.** Copper sequestration inhibits nuclear factor (NF)-κB (A) which reduces the expression of proangiogenic growth factors VEGF (vascular endothelial growth factor) and bFGF (bFGF, basic fibroblast growth factor) and inhibits angiogenesis (B). TTM affects extracellular lysyl oxidase (LOX) (C), Cu/Zn superoxide dismutase (SOD1) (D), and mitochondrial cytochrome *c* oxidase (CcOx) (E) enzyme activity. Inhibiting production of growth factors and cytokines, e.g., transforming growth factor beta (TGF-β) and tumor necrosis factor alpha (TNF-α), underlies the anti-fibrotic and anti-inflammatory properties of TTM (F). Cu, copper; H_2_O_2_, hydrogen peroxide; ΔΨm, mitochondrial membrane potential; TTM, tetrathiomolybdate.

**Fig. 5:**
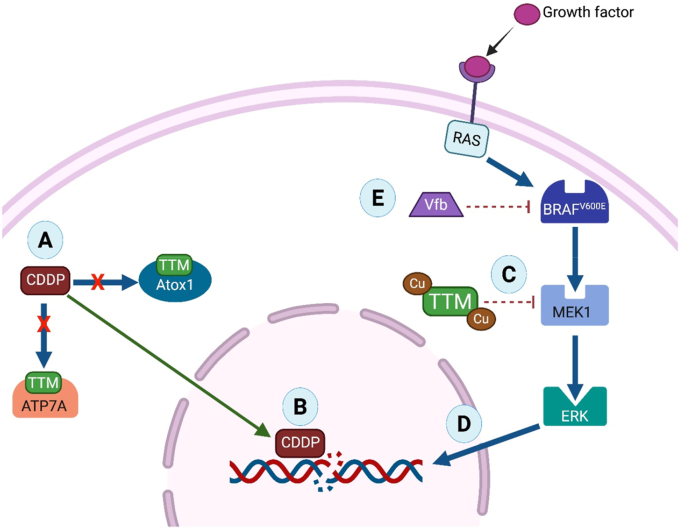
**Alternative anticancer mechanisms with TTM treatment.** Intracellular copper transport proteins (Atox1 and ATP7A/B) mediate cellular efflux of cisplatin (CDDP) (A). Binding of TTM to these proteins increases intracellular cisplatin level by reducing its efflux (B). TTM also inhibits the activity of MAPK kinase kinase-1 (MEK1) (C) and MAPK/ERK-induced cell proliferation (D). This enhances the effects of BRAF-kinase inhibitors (e.g., vemurafenib; Vfb), which inhibit the MAPK pathway to suppress the progression of BRAF^V600E^-induced cancers (E). Atox1, antioxidant 1 copper chaperone; ATP7A, ATPase copper transporting alpha; ERK, extracellular signal-regulated kinase; RAS, rat sarcoma.
(iii) Inflammation and fibrosis
  The interaction between copper and the transcription factor NF-κB provided the initial rationale for investigating the utility of TTM in treating fibrotic and inflammatory conditions (Fig. 4A–F). Promising results were obtained in mouse models of pulmonary [114] and hepatic [115,116] fibrosis. Significant reductions in pro-inflammatory cytokines (TNF-α, [interleukins] IL-1β, IL-2, IL-6) and fibrosis-associated factors (transforming growth factor-beta; TGFβ, and LOX) were observed with TTM in numerous reports studying inflammatory phenotypes [[116], [117], [118], [119], [120], [121], [122], [123], [124], [125], [126], [127], [128]]. Attenuating the production and activity of proinflammatory cytokines may explain the protective effects of TTM in animal models of arthritis [129,130] atherosclerosis [126] and psoriasis [131].
  A double-blind trial compared ATTM against standard of care in patients with primary biliary cirrhosis [132]. Predefined primary endpoints for efficacy (impact on two liver function tests and one proinflammatory cytokine) were achieved and the drug was well tolerated. Two further studies include a Phase 3 study that investigated ATTM for primary biliary cirrhosis and a Phase 1/2 safety study in idiopathic pulmonary fibrosis patients; the results of these trials remain unreported [133,134]. The translational impact of this approach is so far inconclusive.
(iv) Neurodegenerative conditions
  Neurodegenerative conditions, including Alzheimer's disease (AD) and Parkinson's disease (PD), are characterized by several molecular hallmarks, including misfolded protein aggregation (e.g., amyloid-β [Aβ] and α-synuclein), mitochondrial dysfunction, oxidative stress, and metal ion dyshomeostasis, particularly involving copper and iron. In AD, excess copper interacts with Aβ, accelerating its aggregation into oligomers and plaques [135]. In addition, redox-active Cu–Aβ complexes catalyze the production of reactive oxygen species (ROS). The resulting oxidative damage contributes to synaptic dysfunction and neuronal loss [136]. Accordingly, copper chelation has been investigated as a potential strategy to mitigate disease progression [137].
  In an AD transgenic mouse model, ATTM treatment significantly reduced cortical Aβ accumulation and improved learning deficits when administered orally for three months [138]. Treatment-dependent cognitive improvements in these mice suggest that ATTM can mitigate AD-related pathology. Further evidence showed that ATTM promoted the non-amyloidogenic processing of amyloid precursor protein (APP) by upregulating ADAM10 expression via melatonin receptor-dependent signaling pathways [139]. This shift reduced Aβ production while increasing the release of soluble APPα, a neuroprotective fragment known to improve synaptic function.
  TTM has sulfide-releasing properties described in more detail below. These may be relevant in neurodegenerative diseases as plasma sulfide levels are reduced in patients with AD, vascular dementia, and cerebrovascular disease, and correlate negatively with AD severity [140]. Supporting this rationale, sulfide administration to a transgenic mouse model reduced beta amyloid (Aβ) accumulation and improved spatial memory via the phosphatidylinositol 3-kinase/protein kinase B (PI3K/Akt) pathway activation [141]. Sulfide also inhibited tau hyperphosphorylation through persulfidation of glycogen synthase kinase-3β (GSK-3β), a key tau-regulating enzyme [142]. Sulfide donors also protected against homocysteine-induced blood-brain barrier disruption and synaptic dysfunction in rodents [143].
  Conversely, a recent report in Alzheimer's disease and related dementia (ADRD) patients reported elevated acid-labile and bound sulfide species, correlating with cortical thinning and cognitive decline [144]. Authors proposed sulfide overproduction may act as a cytoprotective response to oxidative stress, but that accumulation of bound and acid-labile sulfide present potentially injurious by-products of this response. Elevated sulfide has also been reported in other disorders, such as in ALS patients’ cerebrospinal fluid [145], where ATTM-derived sulfide may be contraindicated. A deeper understanding of sulfide pool dynamics is needed to clarify whether they contribute to pathology or therapeutic benefit, and to guide rationale for investigation of TTM in these conditions.
  While clinical studies using ATTM in AD are lacking, trials using other copper-chelating agents (e.g., PBT2) have investigated the potential of this approach. In a Phase II trial, PBT2 reduced cerebrospinal fluid Aβ levels and improved cognitive function over 12 weeks but did not affect plasma biomarkers of AD or serum copper/zinc levels [146,147]. In other neurodegenerative conditions, metal ion chelation has also shown mixed results. In a Phase II clinical trial in Huntington's disease, PTB2 did not improve cognitive improvement [148], and use of deferiprone in PD patients even suggested detrimental effects [149]. These findings underscore the complexity of targeting metal (dys)homeostasis in neurodegenerative conditions and suggest the need for approaches that go beyond metal chelation.
(v) Ischemia-reperfusion injury (IRI)

Modulation of copper homeostasis was considered the foremost pharmacological mechanism of action of thiomolybdates until our group [150,151] and others [152] reported their activity as slow-release sulfide donors.

Hydrogen sulfide comprises H_2_S gas and anionic hydrosulfide (HS^−^); unless stated otherwise, both forms are referred to herein as sulfide. Like TTM, sulfide has a complex history. It has long been considered an environmental pollutant but is now also recognized as an important physiological mediator [153] and putative therapeutic [154]. Sulfide is classified as the third endogenous gasotransmitter alongside nitric oxide and carbon monoxide [155]. The *in vivo* metabolic effect of exogenous sulfide delivery was first demonstrated using inhaled H_2_S to induce a profound and reversible ‘suspended animation-like state’ in mice [156]. This could protect against acute circulatory insults in mice [157,158] but could not be translated to larger mammals [159,160]. In a Phase 2 clinical trial the basic salt, sodium sulfide (Na_2_S; IK-1001), was administered intravenously to patients undergoing coronary artery bypass graft surgery; the study was abandoned, and trial results remain unpublished [161].

The mechanism of action of sulfide to induce ‘suspended animation’ is its ability to rapidly reduce mitochondrial respiration through inhibition of CcOx [162]. Sulfide toxicity can however arise at high dose from excessive CcOx inhibition [[162], [163], [164]]. Conversely, at lower concentrations sulfide is cytoprotective, showing efficacy across myriad pathologies, and supports mitochondrial respiration by acting as an electron donor through its oxidation by sulfide-quinone oxidoreductase [165].

Due to the very rapid sulfide release from basic sulfur salts and challenges with targeted delivery, focus shifted to slow(er) release sulfide donors; numerous classes now exist [166]. Liberation of sulfide from TTM was previously hypothesized and estimated in an *in vitro* study using a potassium salt [167]. We additionally characterized BCTTM and, within the broader thiometallate drug class, the tungsten analogue of ATTM, ammonium tetrathiotungstate (ATTT; [NH_4_]_2_WS_4_), using *in vitro* (sulfide release) and *in vivo* (rodent pharmacokinetic/pharmacodynamic; PK/PD) assays [168].

**Fig. 6:**
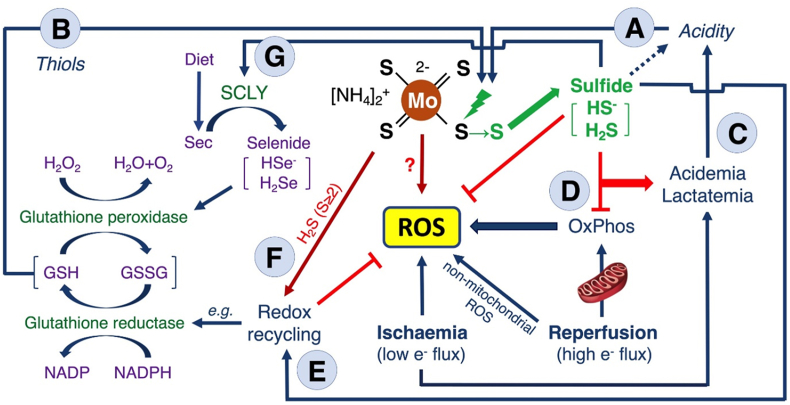
**Overview of putative TTM mechanisms in ischemia/reperfusion injury.** Acidic conditions (A) and oxidized or reduced thiols (B) promote liberation of sulfide from TTM. Acidemia and lactatemia are biomarkers of sulfide pharmacodynamic activity (C). During reperfusion, TTM-derived sulfide slows electron flux and decreases overproduction of mitochondria-derived reactive oxygen species (ROS) (D). TTM-derived sulfide may be involved in the recycling of redox-active molecules (E); a direct role through per/polysulfide donation is additionally postulated (F). TTM increases selenoprotein activity, likely through increased selenocysteine gamma-lyase (SCLY) activity that generates hydrogen selenide from selenocysteine (Sec) (G). e^−^, electron; GSH, reduced glutathione; GSSG, oxidized glutathione; H_2_O_2_, hydrogen peroxide; H_2_S/HS^−^, gaseous sulfide/hydrosulfide anion; H_2_Se/HSe^−^, gaseous selenide/hydroselenide anion; H_2_S (S ≥ 2), per/polysulfide; NADP, nicotinamide adenine dinucleotide phosphate (oxidized); NADPH, nicotinamide adenine dinucleotide phosphate (reduced); OxPhos, oxidative phosphorylation.

Thiomolybdates offer several favorable properties including linear release of sulfide over time in a pH- and temperature-dependent manner, consistent with hydrolysis of metal-sulfur bonds, and thiol-dependent release, regardless of thiol oxidation status [151,168]. These properties support intracellular sulfide release due to the mildly acidic pH and high concentrations of low molecular weight thiols such as glutathione (Fig. 6A and B). Sulfide release varies across thiometallates depending on both the cation and metal (molybdenum or tungsten) with release from BCTTM and ATTT lower than from ATTM [151,168]. TTM and ATTT uptake into cells occurs partly via the anion-exchanger 1 protein [168]. Inhibiting this transporter in erythrocytes reduced intracellular compound detection and sulfhemoglobin formation. Once inside cells, the mildly acidic and thiol-rich environment favors sulfide liberation close to mitochondria, the intended site of action for putative use in IRI, described below. Of note, ATTM could promptly inhibit oxygen consumption both in isolated muscle fibers and *in vivo* when given intravenously [151]. This indicates a copper-independent mechanism since systemic copper depletion following daily oral administration of TTM takes several weeks [48]. Moreover, non-sulfur copper chelators had no acute metabolic impact.

Various studies have highlighted a role for TTM in mitigating reactive oxygen species toxicity by decreasing mitochondrial membrane potential and thus excessive mitochondria-derived reactive oxygen species production [151,[169], [170], [171]]. ATTM treatment could prevent oxidative cell death *in vitro* in cytotoxicity assays triggered by copper oxide nanoparticles [169], H_2_O_2_ [152], and during IRI [151]. In other studies, ATTM enabled nuclear factor erythroid 2-related factor 2 (NRF2) pathway activation, thereby protecting against arsenic-induced cardiovascular toxicity and cisplatin-induced acute kidney injury [170,171].

Conversely, ATTM could inhibit mitochondrial CcOx (decreasing ATP and mitochondrial membrane potential), yet a paradoxical increase in ROS production was observed, with impacts on cellular functionality in an antioxidant-sensitive manner [172]. TTM has been identified as a SOD1 inhibitor [11] that can be deployed in a titratable manner *in vivo* [173,174]. These studies highlight that the impact of TTM on redox status (yielding either pro- or antioxidant effects) is context specific, with potential activation of opposing mechanisms providing an important consideration concerning its therapeutic use in oxidative pathologies. While experimental evidence is currently lacking, thiometallates likely lie on a redox knife edge [168], with highly oxidized metal centers bound to electron-rich ligands that can interact with other compounds or biological targets in a variety of redox states. No studies have yet investigated the direct effects of TTM on reactive species and thus its potential utility as an antioxidant through ROS scavenging (Fig. 6).

Temporarily constraining oxidative phosphorylation with sulfide triggers a greater reliance on glycolytic ATP thereby inducing hyperlactatemia, while both glycolysis and net ATP hydrolysis release H^+^ ions generating a metabolic acidosis [151,168]. These effects can be utilized in PK/PD studies (Fig. 6C).

The sulfide-donating properties of thiomolybdates has been examined as a potential therapeutic for ischemia-reperfusion injury (IRI). Ischemia occurs when the oxygen and nutrient supply to an organ or tissue bed (regional, e.g., myocardial infarction, stroke) or whole body (e.g., major hemorrhage, cardiac arrest) becomes limited [175,176]. Rapid restoration of perfusion through revascularization or resuscitation is vital to prevent further ischemic injury, however this causes paradoxical damage termed reperfusion injury through excessive production of reactive oxygen species [176]. Taken together, this is known as IRI. In varied rodent and porcine IRI models, ATTM given at the onset of reperfusion, thereby mimicking the clinical scenario, reduced infarct size following myocardial infarction and stroke and improved survival following severe hemorrhage [151,177,178]. The drug was well tolerated with minimal clinical/biochemical events; off-target effects included a transient (1 min) fall in blood pressure, commonplace with sulfide-releasing drugs [179], and skin discoloration (due to red coloration of the molecule) that resolved within 24 h [178].

The mechanisms of therapeutic benefit conferred by ATTM in reperfusion injury are likely multifactorial. During ischemia, cells decrease their metabolic demand to cope with limited oxygen and nutrient supply [180]. On revascularization, these cells receive an abundance of substrate (relative to demand) that drives mitochondrial respiration to produce large quantities of ROS [181]. Excess ROS production is countered by antioxidant defences but depletion of these antioxidants, as seen in IRI, enables damage from reactive species [176]. For instance, in the presence of NO, superoxide forms peroxynitrite (ONOO^−^), while H_2_O_2_ reacts with ferrous or cuprous ions via Fenton (or Fenton-like) reactions to generate ^•^OH which causes indiscriminate damage to nearby biomolecules [182]. Irrespective of the source, excess ROS causes oxidative damage to lipids, proteins, and DNA, with subsequent opening of the mitochondrial permeability transition pore and potential initiation of cell death pathways.

Slowing reactivation of the electron transport chain or decreasing its overactivity with sulfide reduces electron flux, and thus mitochondrial ROS production and oxidative damage (Fig. 6D). Decreasing oxidative metabolism and ROS production through targeted temperature control (therapeutic hypothermia) in patients suffering stroke or head injury have failed to improve outcomes in recent large multi-center studies, likely due to the 6+ hours needed to reach target temperatures [183], by which time tissue damage may have become irreversible. A pharmacological means of safely reducing metabolism (and ROS production) could be more rapidly implemented and potentially more effective [151,178]. Through ATTM additionally acting as a polysulfide donor, the potential generation of powerful reducing molecules (e.g., glutathione persulfide; GSSH) may contribute to intracellular redox recycling (and cytoprotection) in cells where antioxidant defenses are strained (Fig. 6E and F) [168].

In our porcine myocardial infarction model ATTM increased tissue glutathione peroxidase (GPx) activity [178]. GPx isozymes detoxify peroxides via thiols, with GPx-1 being the most abundant and crucial in myocardial IRI [[184], [185], [186]]. Elevated GPx activity was observed in both ischemic and remote myocardial regions in our model, indicating a systemic drug effect rather than a hypoxia-specific response. Though the underlying mechanism remains unclear, one possibility is sulfide-induced activation of selenocysteine lyase that increases hydrogen selenide production, enhancing synthesis of selenium-containing GPx (Fig. 6G) [187].

A further antioxidant mechanism sensitive to sulfide treatment is the Kelch-like ECH-associated protein 1/nuclear factor erythroid 2-related factor 2 (KEAP1/NRF2) signaling pathway. Under physiological conditions, KEAP1 constrains NRF2 activity by ubiquitination and proteosomal degradation [188,189]. In response to stressors (e.g., oxidative stress) detected by KEAP1 cysteine sensors, NRF2 evades ubiquitination, and translocates to the nucleus where it promotes transcription of numerous antioxidant genes [190]. TTM induced NRF2 activation through a mechanism involving AMP-activated protein kinase/mammalian target-of-rapamycin (AMPK/mTOR) signaling and autophagic degradation of KEAP1 via Cys151 [171]. Using the sulfide donor, GYY4137, persulfidation of KEAP1 could also invoke this response via Cys151 [191]. This same cysteine sensor was linked to NRF2-driven cardioprotection in earlier sulfide studies [192].

## 5 Limitations and developmental perspectives

The intended pharmacological action of TTM, either as a copper chelator or sulfide donor, can be achieved by the route of administration and duration of treatment. Copper chelator effects follow chronic oral dosing (over 4–6 weeks) whereas intravenous administration can utilize its sulfide donor properties in acute care scenarios. Further investigation into the overlap of these mechanisms is warranted, particularly where opposing effects are anticipated. There are contexts where copper chelation and sulfide liberation could be preferable, for example in treating inflammatory or fibrotic conditions. Conversely, the copper chelating or SOD-inhibiting effects of TTM may be unfavorable e.g., in ischemia-reperfusion injury, while the pro-angiogenic effects of sulfide may be considered undesirable in the tumor microenvironment.

Pharmaceutical development of thiomolybdates is not straightforward. Later investigations in Wilson's disease have used the bis-choline salt which is more stable due to the nature of the cation [80]. Indeed, the copper chelating abilities of TTM require the anionic component of the molecule (MoS_4_^2−^) to be intact – i.e., without or before the liberation of sulfide that yields mixed oxothiomolybdate species e.g., MoO_2_S_2_^2−^ [37]. This is important for long-term storage, with aspects of the purification process and vialling potentially impacting on the integrity of solid-state material, and thus shelf-life. For example, during the chemical development of ATTM, the sulfide release profile is altered by conditions such as air contamination and moisture (derived from residual solvent), even at levels close to the detection limit using sensitive analytical tools (unpublished data). Failure to constrain these factors within appropriate limits leads to material that increases its sulfide release profile over time, even in unopened, argon-packed vials, and thus variations in potency *in vivo*. Researchers should establish sulfide release assays for testing all new batches of thiomolybdate materials and set standards/limits if used for this purpose. Residual moisture also causes polymerization of solid-state material to aqueous insoluble species such as Mo_2_S_8_ or Mo_2_S_7_ (+H_2_S/HS^−^) which hinders rapid dissolution in time-critical scenarios such as revascularization of ischemic organs. Next-generation thiomolybdates intended for use as sulfide donors should exhibit improved solid-state stability, a rapid dissolution profile suited to time-critical scenarios and maintained capacity to release adequate bioactive sulfide.

## 6 Conclusion

Context-specific activity of thiomolybdates have shown utility in numerous pathologies. Long-term veterinary and clinical use to treat copper toxicity and investigation in humans as an anti-cancer agent have demonstrated a good safety profile with adverse effects manageable by dose reduction. More recent investigations suggest the utility of thiomolybdates as adjunct therapies alongside chemotherapeutic drugs or during the revascularization of ischemic organs, acting through multiple putative or established modes of action. These findings support further development as either first in-class or next-generation therapeutics across several disease areas, with a cautionary note that potentially opposing mechanisms of action warrant further investigation.

## CRediT authorship contribution statement

**Yihan Wu:** Writing – original draft, Visualization. **Khalid S. Alotaibi:** Writing – review & editing, Visualization. **Kevin Yu:** Writing – original draft, Visualization. **Tom Durham:** Writing – review & editing. **Felipe Dal-Pizzol:** Writing – review & editing. **Mervyn Singer:** Writing – review & editing. **Alex Dyson:** Writing – review & editing, Writing – original draft, Visualization, Supervision, Project administration, Conceptualization.

## Funding statement

Not applicable.

## Declaration of competing interest

Further to submission of the manuscript ‘*Mechanisms, therapeutic uses, and developmental perspectives of redox-active thiomolybdates’* for consideration of publication in Redox Biology, the authors confirm that:

Alex Dyson and Mervyn Singer hold intellectual property and are developing thiomolybdates for the treatment of ischemia/reperfusion injury.

All other others declare that they have no conflict of interest.

## Data Availability

No data was used for the research described in the article.
