# Supplementary material for: Mechanisms, therapeutic uses, and developmental perspectives of redox-active thiomolybdates
Source: Redox Biol. 2025 Aug 27;86:103846. doi: 10.1016/j.redox.2025.103846 (PMC12445612; doi:10.1016/j.redox.2025.103846)
Supplement: Multimedia component 1 [file mmc1.docx]

*Supplementary Table 1: Interventional clinical trials investigating use of tetrathiomolybdate for Wilson’s disease.*

| **Study type** | **Treatment (study length)** | **Sample size (n)** | **Key results** | **Adverse effects/ other comments** | **References** |
| --- | --- | --- | --- | --- | --- |
| Phase 2 (open label) | ATTM (8 weeks) + Zinc maintenance therapy or SoC. | 55 | Prevention of neurological deterioration in 4% of ATTM-treated patients vs 50% for SoC. | Anemia, leukopenia, aminotransferase elevations. | [27,45,46] |
| Phase 3 (double- blind RCT) | ATTM or trientine (8 weeks) + Zinc. | 48 | Prevention of neurological deterioration in 4% of ATTM-treated patients vs 17% for trientine. | Anemia, leukopenia, aminotransferase elevations. | [47] |
| Phase 2 (dose-finding study) | ATTM (8 or 16 weeks) + Zinc. | 40 | Less control of free copper with ATTM compared with earlier Phase 2 study due to change in dose frequency. Neurological function deterioration in 15-20% of patients. No difference in efficacy reported in current study between treatments regimens. | Significant reduction in incidence of anemia/ leukopenia and elevated transaminase levels. | [48] |
| Phase 2 (open label) | BCTTM (24 weeks) | 28 | Significant decrease in non-ceruloplasmin (free) copper (NCC) corrected for TTM-complexed copper. Once daily dosing. | Increased liver transaminases. Serious adverse events reported in <4% of patients. | [50] |
| Phase 3 (rater- blinded RCT) | BCTTM or SoC (48 weeks)  2:1 in favor of active treatment | 214 | Superior control of copper status, reported as AUEC NCC (0-48 weeks). Unified WD rating scale showed no statistical difference between groups. | Increased alanine aminotransferase in 14% of patients. Optional continuation terminated by sponsor (NCT03403205). | [10] |
| Phase 2 (rater-blinded) | BCTTM (48 weeks) | 31 | Assessed liver biopsies to evaluate copper concentration/histo-pathologic changes. BCTTM reduced intestinal copper uptake but did not facilitate biliary copper excretion. | Increased transaminases. | [51] |

*ATTM, ammonium tetrathiomolybdate; AUEC, area under the effect curve; BCTTM, bis-choline tetrathiomolybdate; RCT, randomized clinical trial; SoC, standard of care; WD, Wilson’s disease.*

*Supplementary Table 2: Interventional clinical trials investigating use of tetrathiomolybdate for treatment of cancer*.

| **Study type** | **Drug and Cancer type, (study length)** | **Sample size (n)** | **Key results** | **Adverse effects/ other comments** | **Reference** |
| --- | --- | --- | --- | --- | --- |
| Phase 1 (open label) | ATTM;  Various metastatic cancers  (90 days) | 18 | Serum ceruloplasmin (Cp) is feasible indicator of copper status. ATTM effectively depletes copper status in cancer patients. | Mild, reversible anemia observed in four patients with Cp levels between 10 –20% of baseline. | [104] |
| Phase 2 (open label) | ATTM;  Metastatic kidney cancer  (12 weeks to first timepoint) | 15 | Effective copper depletion in all patients. Stable disease for at least 6 months (median duration 34.5 weeks) in 4/15 patients. No correlation between serum proangiogenic factors (IL-6, IL-8, VEGF, bFGF) and copper depletion/disease progression. | Grade 1-2 fatigue in almost all patients (one discontinued), sulfur eructation, diarrhea, dizziness, rash, anemia, granulocytopenia. | [112] |
| Phase 2 (open label) | ATTM;  Hormone-refractory prostate cancer (HFPC)  (20 weeks) | 19 | Effective copper depletion in 17/19 patients. ATTM not effective in slowing the progression of HFRP. No correlation of copper depletion with serum IL-6, IL-8, bFGF). No correlation between prostate-specific antigen and levels of angiogenesis factors. | Grade 3-4 toxicity in 8 patients including hematuria, neutropenia, lymphopenia, musculoskeletal pain, unstable angina. NB, lymphopenia also reported before drug initiation. | [107] |
| Phase 1 (open label) | BCTTM;  Advanced solid tumours (8 weeks) | 18 | Decreases in total plasma copper and Cp were dose-dependent, and more rapidly decreased with concomitant proton pump inhibitors. Stable disease in 13 patients for at least 6 months. SOD1 activity decreased in blood cells. No impact on cytokines/growth factors. | Fatigue, sulfur eructation, anemia, neutropenia, lymphopenia. | [11] |
| Phase 2 (open label) | ATTM; Malignant mesothelioma (up to 57 months post-surgery, median = 15 months) | 30 | Effective in reduction of Cp and VEGF. Time to disease progression for Stage I/II patients significantly lower than an historic (1990-2005) cohort receiving cytoreductive surgery. No comparative impact in Stage III patients. | Dizziness, fatigue, granulocytopenia, thrombocytopenia, anemia. Some required red blood cell transfusions. | [111] |
| Phase 1 (open label) | ATTM; Metastatic colorectal cancer  (12 weeks) | 24 | ATTM investigated in combination with irinotecan, 5-flurouracil, and leucovorin (IFL) chemotherapy. Dose intensity of IFL maintained with addition of ATTM. | Granulocytopenia, thrombocytopenia, anemia, diarrhea, sulfur eructation. | [106] |
| Phase 2 (2 dose level, random-ised) | BCTTM; Hormone-naïve prostate cancer  (12 months) | 52 | Cp levels did not correlate with prostate-specific antigen. No dose response observed. | Leukopenia, lymphopenia, anemia, fatigue, diarrhea, rash. | [109] |
| Phase 2 (open label) | ATTM;  Breast cancer  (2 years) | 40 | Reduction of Cp that correlated with decreased circulating epithelial progenitor cell counts and LOXL2 levels. | Leukopenia, neutropenia, anemia, fatigue. | [108] |
| Phase 2 (open label) | ATTM;  Breast cancer  (2 years) | 75 | Effective in reduction of Cp in 91% of triple negative breast cancer patients; 41% and 57% reduction in luminal and *Her*2-positive, respectively. Decrease in epithelial progenitor cell counts but not circulating angiogenesis markers. | Leukopenia, neutropenia, anemia, fatigue. | [105] |
| Phase 1 (open label) | ATTM;  Non-small cell lung cancer | 26 | ATTM administered in combination with carboplatin. Study (NCT01837329) completed in 2019; no results reported. | | [103] |
| Phase 2 (open label) | ATTM;  Breast cancer | 16 | Study (NCT00195091) assessing time to cancer progression with ATTM terminated; PI left the sponsoring institution. | | [110] |
| Phase 1b + Phase 2 (random-ised, open label) | ATTM;  Breast cancer | (up to 186) | Currently recruiting, study NCT06134375 will assess safety (Phase 1b) and efficacy (Phase 2) of (distant) relapse-free survival between TTM and capecitabine versus capecitabine alone in patients with triple negative breast cancer after standard surgery and neoadjuvant chemotherapy. | | [113] |

*ATTM, ammonium tetrathiomolybdate; BCTTM, bis-choline tetrathiomolybdate; bFGF, basic fibroblast growth factor; Cp, ceruloplasmin; Her2, human epidermal growth factor receptor 2; IFL, ‘irinotecan, 5-flurouracil, and leucovorin’; IL, interleukin; LOXL2, lysyl oxidase like-2; SOD1, superoxide dismutase-1; VEGF, vascular endothelial growth factor.*
